# Preparation and Experimental Investigations of Low-Shrinkage Commercial Concrete for Tunnel Annular Secondary Lining Engineering

**DOI:** 10.3390/ma15196848

**Published:** 2022-10-02

**Authors:** Jin Yang, Tie Wang, Xingyang He, Ying Su, Fei Dai, Long Xiong, Rixu Zhao, Xuyang Duan

**Affiliations:** 1 School of Civil Engineering, Architecture and Environment, Hubei University of Technology, Wuhan 430068, China; 2 Building Waterproof Engineering and Technology Research Center, Hubei University of Technology, Wuhan 430068, China; 3 China Construction Ready Mixed Concrete Co., Ltd., Wuhan 430000, China; 4 China Construction Third Bureau First Engineering Co., Ltd., Wuhan 430040, China

**Keywords:** secondary lining engineering, commercial concrete, low shrinkage

## Abstract

Secondary lining concrete is frequently used in underground tunnels. Due to the internal restriction of the annular concrete segment, micro-cracks may be caused by temperature stress and volume deformation, thus affecting the safe transportation of the tunnel. The purpose of this study is to provide a concrete experimental basis with low hydration heat and low shrinkage for tunnel engineering with different construction requirements. Different amounts of expansion agent (EA), shrinkage-reducing agent (SRA), and superabsorbent polymer (SAP) were considered in commercial concrete. It was found that EA elevated the degree of hydration and the hydration exothermic rate, while SRA and SAP showed the opposite regularity. SRA has the optimum shrinkage reduction performance with a 79% reduction in shrinkage, but the strength decreases significantly compared to EA and SAP groups. The effect of the combination of different shrinkage reducing components in commercial concrete is instructive for the hydration rate and shrinkage compensation in secondary lining engineering.

## 1. Introduction

As the global industrialization of the 21st century continues to grow, cities have become the main gathering place for people. In order to meet the growing material and cultural needs of the population, the development of urban infrastructure has become a hot topic [1]. Railways and roads have become an important means of promoting intercity collaboration. However, obstacles such as rivers, lakes and mountains limit the planning and layout of traffic routes and affects the close connection between regions. Therefore, vigorously developing underground rock and soil resources is a new direction of our development. Underground tunnels are built for transport of people and goods, water supply channels and sewage treatment, which have obvious advantages over overground installations [2]. Tunnels can optimize route planning and reduce damage to vegetation while overcoming terrain and other obstacles. Underground transportation can effectively alleviate traffic congestion in cities.

**Figure 1:**
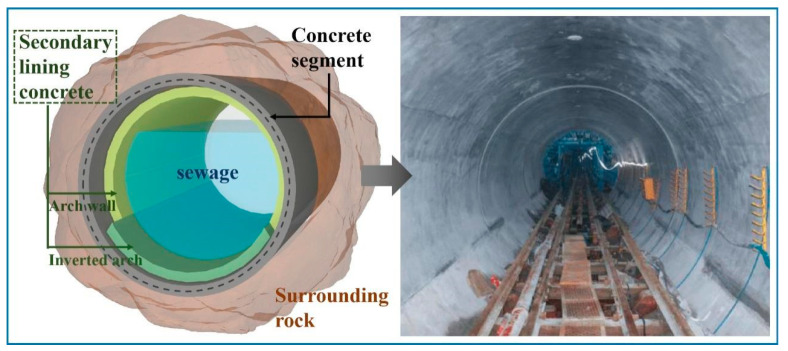
Schematic diagram of secondary lining engineering of underground tunnel.

The design and construction of most tunnels generally follow the New Austrian Tunnelling method [3,4]. For the stability of the surrounding rock and to ensure operational safety, a support structure with sufficient strength is necessary after the subject tunnel body has been excavated. The composite lining structure is mainly composed of two parts: primary support and secondary lining [5]. The primary support mainly serves to bear the load and keep the stabilization of the surrounding rock. The supporting material for the primary support usually consists of concrete and pre-positioned steel bars. The high-fluidity concrete is transported through a closed pipe system and then sprayed onto the exposed rock surface to form support shell. The secondary lining is constructed after the surrounding rock and the primary support are stabilized, generally using reinforced concrete. The secondary lining structure can prevent the deterioration of surrounding rock and the accidental load caused by geological disasters such as earthquakes and enhance the durability of the tunnel structure system. Furthermore, the secondary lining is combined with the primary support structure to share part of the surrounding rock load [6], enhancing the stability of surrounding rock. Figure 1 is a schematic diagram of the second lining project structure.

The volume stability of the secondary lining structure has a prominent impact on the quality and service life of the tunnel. It is reported that the cracking of lining structures is caused by internal and external factors. The failure caused by external factors is mainly because the load acting on the supporting structure exceeds the bearing capacity of the structure [7,8]. Structural cracking caused by internal factors is mainly related to the autogenous shrinkage and temperature stress of concrete materials. Although these cracks do not directly lead to a diminution in the bearing capacity, they can reduce the durability of the secondary lining structure, which in turn has an impact on the safety of the structure [9,10].

At present, the construction of tunnels is mostly carried out in complex geological conditions, which comes up with higher demands for the shrinkage and crack resistance of secondary lining concrete materials. To avoid the occurrence of cracks in secondary lining concrete, many scholars devote themselves to studying the influence of different methods to restrain autogenous shrinkage on the performance of concrete. Lawler et al. [11] and Kaufmann et al. [12] found that the crack resistance of concrete is significantly enhanced with the addition of steel fibers (SFS). Meda et al. [13] studied concrete segments containing steel fibers and glass fibers and showed that this composite fiber increases the maximum bearing capacity and reduces the crack width. In tunnel engineering, according to the construction requirements, the commercial secondary lining concrete needs to be pumped to the designated pouring site.

In addition to fibers, concrete additive can be used to compensate for shrinkage and inhibit cracking of the concrete. Expansion agent is an admixture that can cause a certain volume expansion of concrete. The types of expansion agents are calcium sulfoaluminate, calcium oxide, etc. Among them, the expansion effect of calcium oxide expansion agent is mainly caused by the hydration of calcium oxide crystals to form calcium hydroxide crystals, and the volume increases. This in turn counteracts the strain caused by the shrinkage of the cement-based material. Zhao et al. [14] studied the performance of CaO-based expansion agent (CEA) and MgO-based expansion agent (MEA) on cement-based materials and indicated that the combined addition can optimize the pore structure of mortar. Meddah et al. [15] added shrinkage-reducing agent (SRA) and expansion agent (EA) to high performance concrete, which significantly reduces autogenous shrinkage and increases the limit value of cracking stress. Concrete SRA is an organic compound that curtails the surface tension of pore solution of concrete, which reduces capillary negative pressure and shrinkage stress. Zuo et al. [16] conducted a cement net mortar experiment in which two shrinkage reducers were incorporated into water to cement ratio of 0.3 net mortar and found that the two SRAs reduced early self-shrinkage by 55% and 34%. Bentz et al. [17] explored the early shrinkage of SRA cement-based materials, and the results showed that SRA could maintain internal humidity and reduce autogenous shrinkage under the same hydration time. Super absorbent polymer (SAP) is a functional polymer material. For cement-based building materials, SAP can also be used as a “miniature reservoir” to supplement the internal curing moisture for the “growth” of cement concrete and compensate for the shrinkage of concrete. Almeida et al. [18] demonstrated the significant internal curing effect of SAP in the Portland cement replacement by ground granulated blast-furnace slag (PC-GGBS) system, which was able to observably reduce autogenous shrinkage. According to previous research, EA, SRA, and SAP all have the characteristics of shrinkage and crack resistance. In this paper, different shrinkage-reducing components are used in engineering practice and the shrinkage cracking of a multi-component cementitious material system is used as the research object to supplement the secondary lining concrete engineering. The theoretical basis of shrinking functional components is provided, and the applicability of these shrinkage-reducing components to multi-component cementitious material systems is explored.

For the shrinkage and crack of secondary lining concrete with high volume mineral admixture, this study comparatively analyzed the effects of different shrinkage reducing components on various properties of secondary lining concrete. EA, SRA and SAP were characterized by laser particle size measurement, X-ray fluorescence and SEM, respectively. The effects of three shrinkage-reducing components on the hydration process and compensatory shrinkage of secondary lining concrete with high volume mineral admixture were evaluated by the rheology, compressive strength, autogenous shrinkage, heat of hydration, capillary water absorption, electric flux, and pore structure. This paper provides a reference for the mix proportion design of secondary lining concrete in practical engineering.

## 2. Experimental Programs

### 2.1. Materials

**Figure 2:**
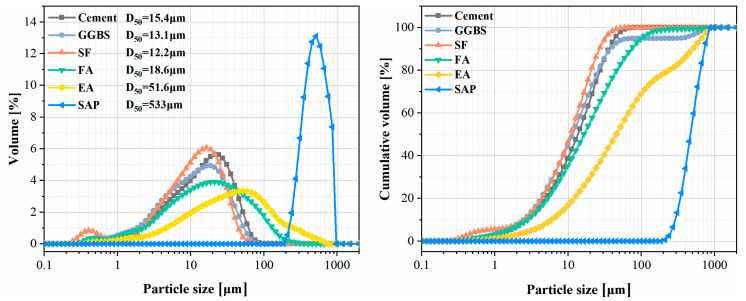
Particle size distribution and cumulative curve distribution of materials.

**Figure 3:**
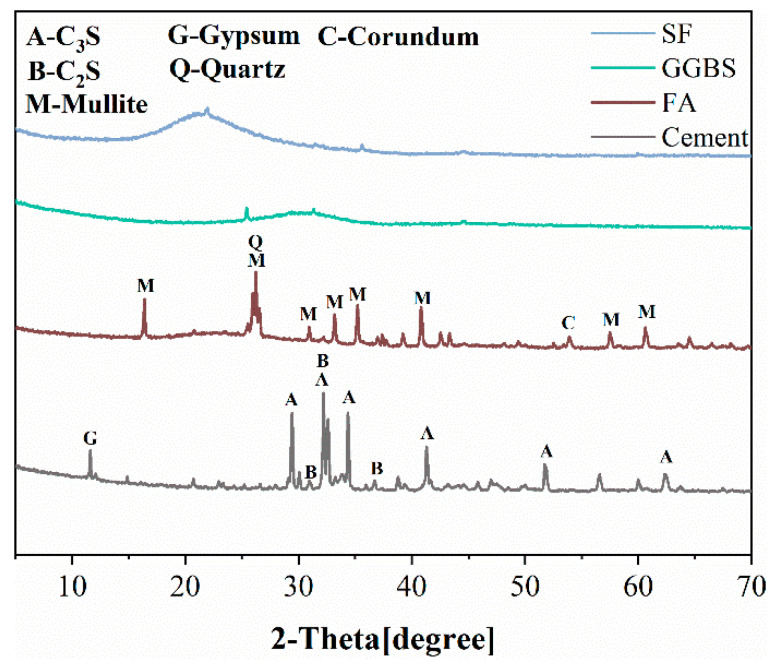
XRD patterns of materials.

This paper uses ordinary Portland cement (P.O. 42.5). The particle size distribution and cumulative distribution of the ground granulated blast furnace slag (GGBS), silica fume (SF) and fly ash (FA) are shown in Figure 2. The chemical composition of the cement, GGBS, SF, FA and EA was determined by X-ray fluorescence (XRF), and the results are shown in Table 1. In addition, the XRD patterns of raw materials are shown in Figure 3. The fine aggregate is well-graded river sand (0–4.75 mm) and the coarse aggregate is natural gravel (5–16 mm); its bulk density is about 1490 kg/m^3^. The polycarboxylate superplasticizer (PCE) is a high-performance water reducer with a water reduction rate of 38%.

The different shrinkage-reducing components used are EA, SRA and SAP. EA is a compound swelling agent, the main components include calcium oxide–calcium sulfate, with a restrained expansion rate at 7 d (volume fraction) in water ≥0.034%. SRA is a solution whose main component is a polyether organic compound. SAP is an acrylic acrylamide copolymer with a particle size of 0.2–1 mm.

### 2.2. Proportions of Concrete Mix

The concrete mix is shown in Table 2, with a water/binder (W/B)of 0.34 and binder:sand:gravel of 1:1.67:1.71. The hydration heat test was carried out according to the concrete mix without the sand and gravel, and mercury intrusion porosimetry without the gravel.

### 2.3. Water Absorption of SAP

The adsorption tests of SAP in various solutions (deionized water, tap water, 0.2 mol/L NaCl solution and w/c 0.3 pore fluid) were carried out by the teabag method [19]. A measure of 5.9 g of NaCl was added into 500 mL of deionized water to prepare a 0.2 mol NaCl solution. The cement slurry was stirred and a suction filter was used to extract the cement filtrate. Four beakers were filled with deionized water, tap water, 0.2 mol NaCl solution, and w/c of 0.3 pore fluid, to completely immerse the tea bag in the solution. The change in SAP quality within 10 min was recorded, the interval was the 30 s at the beginning, and the quality change was recorded every 2 min after 2 min. The excess water on the surface was removed, the mass marked as *m_g_* dry SAP with mass *m_d_* was weighed and added into wet tea bags, and *m_t_* was weighed regularly. Water absorption was determined by averaging three suction tests. The specific formula is as follows:

(1) $$Q_{t}=\frac{m_{t}-m_{g}-m_{d}}{m_{d}}$$

**Figure 4:**
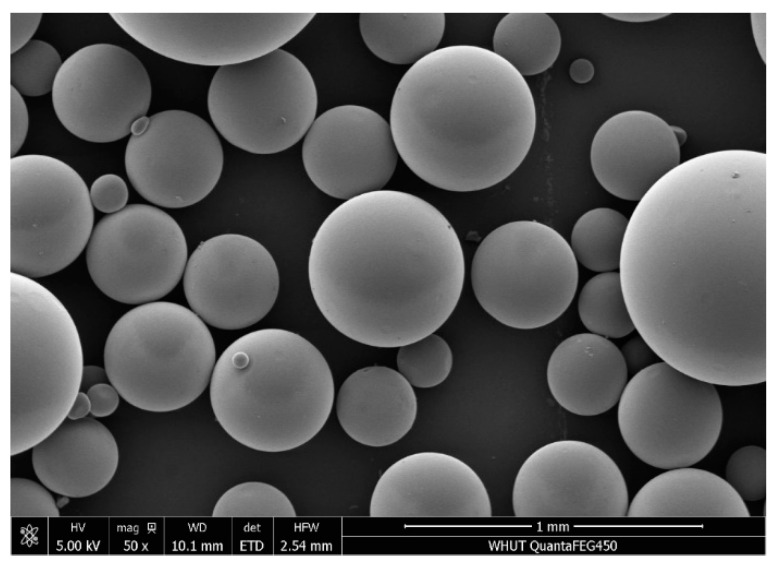
SEM and adsorption kinetics of spherical resin.

The morphology of the SAP under scanning electron microscopy was shown in Figure 4.

**Figure 5:**
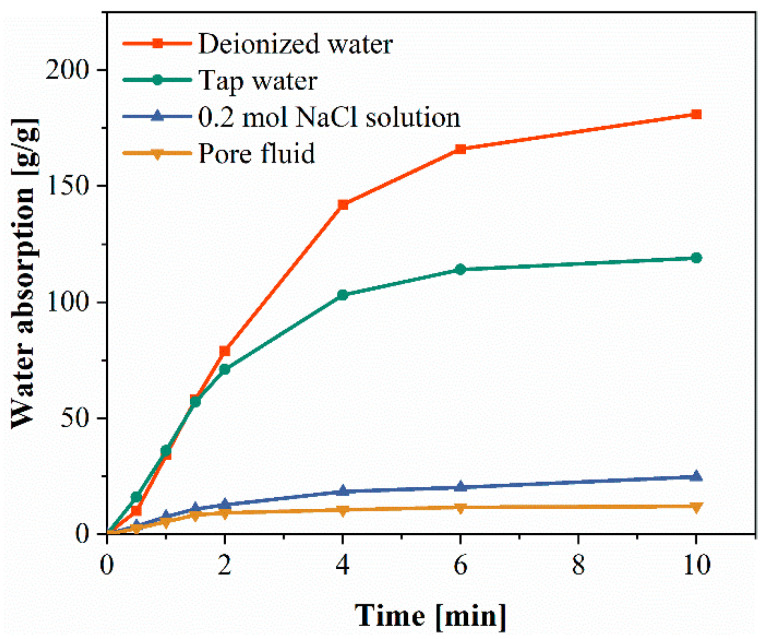
Water absorption of SAP in different liquid environments.

The water absorption of SAP in deionized water, tap water, sodium chloride solution and w/c of 0.3 pore fluid were 180, 110, 20 and 12 times, respectively, according to Figure 5.

### 2.4. Experimental Methods

#### 2.4.1. Rheological Properties

The rheology test was performed using a BROOKFIELD DV3T rheometer. The rate range was 0.01 RPM–1300 RPM, and the torque resolution was 0.15 μN·m [20]. The cement slurry was tested by a rheometer, keeping the room temperature at 25 ± 2 °C. The obtained data were fitted according to the modified Bingham model to obtain the results. Its equation is

(2) $$\tau =\tau_{0}+\eta_{p}\dot{\gamma }+c{\dot{\gamma }}^{2}$$

where *τ* is shear stress (Pa), *τ*_0_ is yield stress (Pa), *η_p_* is plastic viscosity (Pa·s), $\dot{\gamma }$ is shear rate (s^−1^), and *c* is a constant.

#### 2.4.2. Hydration Heat Evaluation

The hydration heat test was carried out by a TAM Air 8-channel heat of hydration microcalorimeter from TA, USA. The microcalorimeter is preheated to the specified temperature before testing, after which the cement paste is stirred well and 6 g of paste is weighed into the bottle. The heat flow and cumulative heat were recorded for 72 h.

#### 2.4.3. Compressive Strength

A 100 × 100 × 100 mm^3^ cube mold was used for the concrete compressive strength test. The fresh concrete mixtures were put up in the standard maintenance room (20 ± 2 °C, relative humidity ≥ 95%). The compressive strength of concrete specimens was tested using the DYE-2000 press with a loading speed of 5 kN/s at 3, 7 and 28 d. The average value of 3 specimens in the same group was regarded as the final compressive strength result.

#### 2.4.4. Autogenous Shrinkage Test

Concrete specimens of 100 × 100 × 500 mm^3^ after 1 d of standard curing were sealed using polyethylene film wraps. The upper and lower surfaces were sealed with a single layer of film to avoid evaporation of internal moisture. The self-shrinkage was detected using the GS-II automatic concrete shrinkage tester produced by Beijing Jinghaiquan Sensing Technology Co., Ltd., Beijing, China. with a range of ±1.0000 mm, and the data were collected automatically.

#### 2.4.5. Capillary Water Absorption

Concrete specimens of 50 mm × Φ 100 mm were cast, cured for 28 d, and then dried in an oven at 60 °C for 2 d. The sides and top of the specimen were wrapped with polyethylene film, sealed with foil tape on the sides, and the underside was sanded with sandpaper. The bottom of specimen was submerged in water by 3 mm. The water absorption quality was observed before 12 h and after 24 h, and the slope of the curve of the water absorption height per unit section was calculated; the square root of time is recorded as the first adsorption coefficient S1 and the second adsorption coefficient S2 [21]. The conversion formula of capillary water absorption S1 and S2 is as follows:

(3) $$A_{t}=\frac{m_{t}-m_{0}}{\alpha \times q}$$

where A_t_ is accumulated water absorption at moment t; m_t_ is the mass of the specimen at moment t; m_0_ is the mass of the specimen after drying; α is the area of the base; q is the density of water.

#### 2.4.6. Chloride Permeability Test

Determination of chloride ion permeability resistance of concrete by electric flux method. Cylindrical concrete specimens with dimensions of 50 mm × Φ84 mm were prepared according to ASTM C1202.

The test block after standard curing for 28 d was immersed in water for 2 d. After curing for 27 d, the blocks were soaked in clean tap water for 24 h and were fixed in the electrical flux tester that the cathode side was 3 wt% NaCl solution, while the anode side was 0.3 M NaOH solution. At room temperature, a voltage of 60 V was applied to the specimen, and the current was recorded every 30 min for 6 h. The average of the three blocks is the final result.

#### 2.4.7. Mercury Intrusion Porosimetry (MIP)

The pore structure of 28 d mortar specimens was tested using a PoreMaster-60 [22]. The samples with a 4–5 mm particle size were dried for 4 h at 50 °C. The samples were first intruded in a low-pressure porosimeter with a pressure up to 3.5139 KPa, and then moved in a high-pressure porosimeter with pressure up to 413,400 KPa. The contact angle between the paste and the mercury was chosen as 130°. The measurable pore size range is 4.5–200,000 nm.

## 3. Results and Discussion

### 3.1. Rheological Property

**Figure 6:**
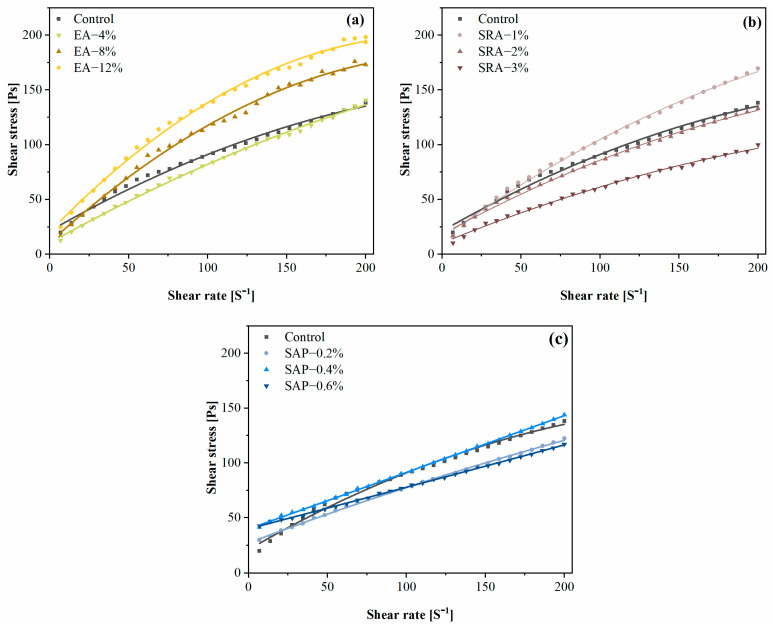
Rheological curves of cement pastes with different shrinkage-reducing components: (**a**) EA (**b**) SRA (**c**) SAP.

Figure 6 shows the rheological curves of cement paste containing different shrinkage-reducing components. The Modified Bingham Model was used to fit the rheological curves of each of the three shrinkage-reducing components, and the results are shown in Table 3.

It is shown that the shear stress and plastic viscosity increase with an increasing dose of EA. The CH crystal formed by the reaction of CaO and water occupies the pores, which increases the friction between particles, thus increasing the shear stress and plastic viscosity [23].

It is worth noting that the yield stress of the SRA group is lower than that of the control, and the plastic viscosity of SRA mixed slurry decreases with the increase in SRA content. This is attributed to the fact that the polar unit of SRA reduces the surface tension and interfacial energy of the pore solution; SRA can improve the dispersion of cement particles as well [24,25].

Although additional water was introduced to compensate for the water uptake of the SAP, this change in the total water–cement ratio would have some different changes with the addition of SAP. The yield stress of the SAP mixed slurry gradually increased, but the plastic viscosity decreased, compared with the control. The increase in the SAP content increases the yield stress, which may be related to the effect of the expanded SAP size on the slurry. Compensated by the additional introduction of water, it affects the overall W/B and reduces the viscosity of the slurry [26].

After comparison, it is concluded that SAP is more suitable for long-distance pumping in engineering, while EA and SRA are more suitable for short-distance pumping.

### 3.2. Hydration Heat

**Figure 7:**
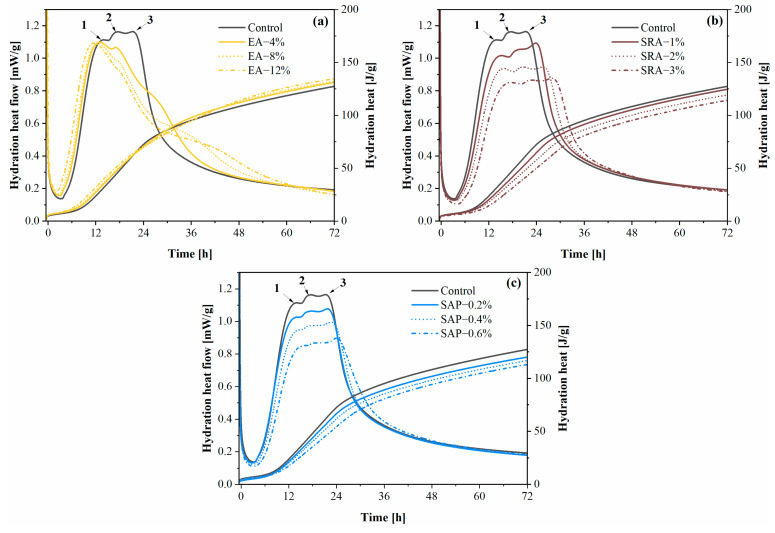
Hydration heat kinetics of pastes with different shrinkage compensation admixtures: (**a**) EA (**b**) SRA (**c**) SAP.

The process of hydration of the cement-based materials with high volume content of FA, GGBS and SF in this study was divided into the initial period, the induction period, the acceleration period, and the retardation period [27,28,29]. The hydration heat flow and hydration heat of different shrinkage-reducing components are shown in Figure 7. Interestingly, three consecutive hydration heat peaks are observed in the control group [30,31,32,33], which are related to the hydration of different mineral admixtures. According to previous studies, cement has a faster hydration rate compared to the mineral admixtures, while the combination of FA and SF tend to have a faster hydration rate than GGBS among the mineral admixture concrete in this paper. Peak 1 is attributed to the hydration heat flow peak of the silicate clinker, which occurs within approximately 12 h [34]. Peak 2 occurs at the 17 h during acceleration period, which was mainly relevant to the hydration of FA and SF [35,36,37]. Peak 3 located at around 21 h of hydration is formed by the hydration of GGBS [37,38].

Figure 7a corresponds to the graph of hydration heat flow and hydration heat after EA incorporation. It is obvious that the effect of EA on the heat of hydration is different from that of SRA and SAP, which is attributed to the chemical effect of EA. One can observe that the addition of EA makes peaks 2 and 3 disappear, while peak 1 is much higher than the peaks doped with SRA and SAP. This is due to the ability of CaO and CaSO_4_ in EA to react with FA, SF and GGBS in advance to form C-S-(A)-H gel and AFt without relying on CH produced by cement hydration [39,40]. The additional chemical reaction of EA with the admixture also makes the heat of hydration higher than that of the control [41]. On the other hand, the additional AFt generated by the reaction of EA results in higher peaks of AFt to AFm conversion in the later stages of hydration [42,43].

In Figure 7b, SRA slowed down the hydration rate of cement and caused the cumulative heat release to decrease with the increase in SRA dosage. The influence of SRA on the early hydration performance of cement slurry was caused by two aspects. On the one hand, SRA molecules formed by covalent bonding of hydrophilic head molecules and hydrophobic tail molecules will reduce the contact area between water and cement particles [44]. With the increase in SRA incorporation, the SRA molecules in solution negatively affect the cement particles and hydration products, the interfacial energy decreases, and the hydration reaction was delayed and decreased [25,45]. On the other hand, the hydrophilia of SRA changed the pore solution polarity [46], reducing the alkaline ion solubility and limiting the hydration of C_3_A in solution [16].

Figure 7c shows the hydration heat flow rate and cumulative heat release of the cement paste incorporated SAP. Obviously, the peak of heat flow was delayed and the cumulative heat release decreased in the SAP group. The SAP adsorbed the alkaline ions produced by cement hydration [47] and gradually released water during further cement hydration, increasing the effective water–cement ratio and decreasing the hydration rate [48]. Predictably, the cumulative hydration heat of the SAP group will be more than the control due to the introduction of more water.

EA can accelerate hydration and promote the hydration process. The difference is that SRA and SAP can significantly reduce the peak heat of hydration and the exothermic rate of hydration, providing a solution to reduce the thermal stress of mass concrete.

### 3.3. Compressive Strength

**Figure 8:**
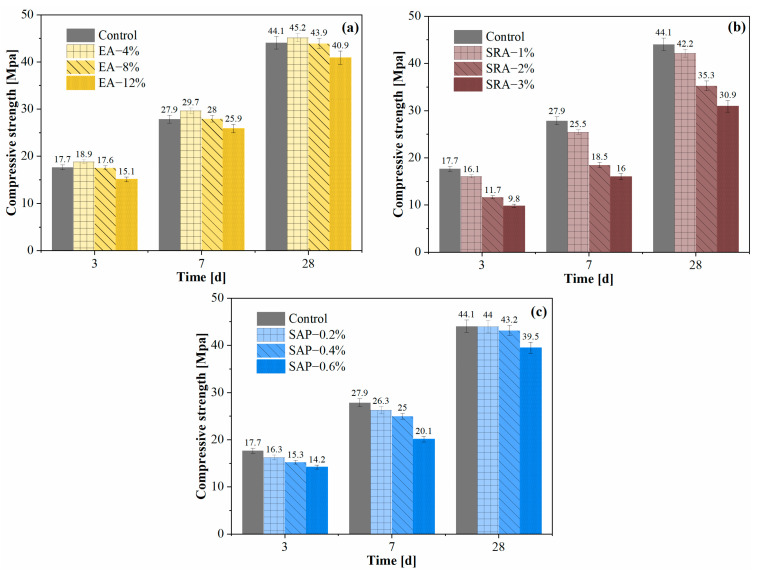
Compressive strength of secondary lining concrete at 3 d, 7 d and 28 d: (**a**) EA (**b**) SRA (**c**) SAP.

The compressive strengths of secondary lining concrete at 3 d, 7 d and 28 d are shown in Figure 8. From Figure 8a, we can see that the compressive strength of the 4% EA-added concrete increases, compared with control at all ages (3 d, 7 d and 28 d). However, the compressive strength of EA-12% EA decreases by 10% at the age of 28 d. The expansion components such as CaO in EA reacted to form Ca(OH)_2_ crystals, which increases in volume and generate expansion force that is transferred to the surrounding cement hydration products [49], optimizing the microstructure of concrete and improving early strength. In addition, higher incorporation of EA will hydrate to generate more CH, which will bring high crystallization pressure and lead to excessive expansion, and the microstructure of concrete tends to loosen, thus negatively affecting the mechanical properties of concrete [49,50].

Figure 8b shows the effect of different amounts of SRA on the compressive strength. The SRA harms the strength of concrete, either at an early or late stage. The decrease in the compressive strength of concrete is attributed to the increase in the SRA content. This is mainly because the incorporation of SRA hinders the hydration reaction of cement and reduces the alkalinity of the reaction system, resulting in a decrease in strength [51]. This result can be confirmed by the heat of hydration test in Section 3.2.

Figure 8c shows that the incorporation of SAP reduces the early compressive strength of concrete. The decrease in early strength after adding SAP is mainly due to two reasons. Firstly, the volume expansion of SAP in concrete due to water absorption reduces the overall strength of concrete. Secondly, SAP increases the effective water–cement ratio of cement paste, which is related to water release. However, SAP has two different effects on the compressive strength of concrete in the later ages: (1) SAP shrinks and leaves macroscopic pores after water release; (2) SAP promotes cement hydration due to water release, forming the dense interfacial transition zone (ITZ) [52]. The small amount of SAP offsets the negative effect of macroscopic pores on the mechanical properties of concrete through internal curing, and the compressive strength of SAP-0.2% is basically the same as that of the control at 28 d.

The analysis of the compressive strength test shows that EA incorporation shows an increase in strength, compared to SRA and SAP. EA is able to improve the strength performance of concrete, and SAP has a negative impact on the early strength, which can be compensated for the previous loss by curing at a late stage. Overall, EA and SAP have small strength loss in engineering practice.

### 3.4. Autogenous Shrinkage

**Figure 9:**
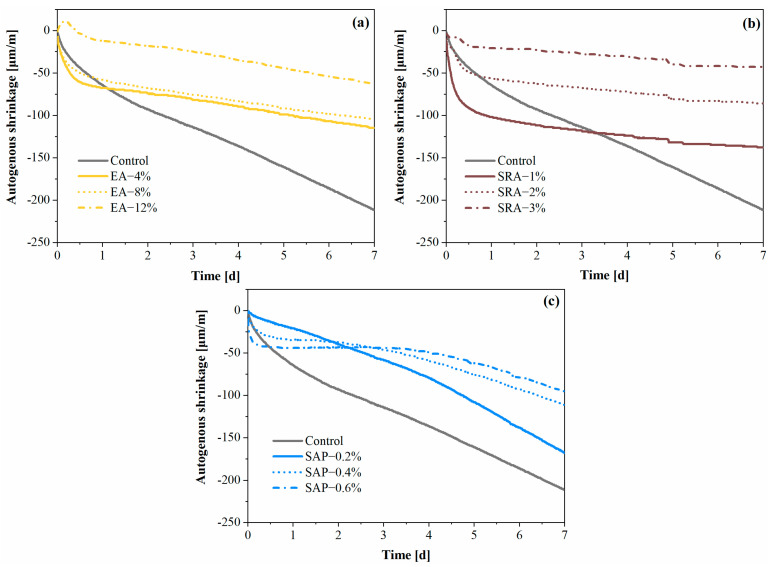
Autogenous shrinkage of secondary lining concretes: (**a**) EA (**b**) SRA (**c**) SAP.

As shown in Figure 9, different shrinkage reduction components can reduce the autogenous shrinkage of concrete. It can be seen from Figure 9a that the concrete with EA was effective in reducing the autogenous shrinkage of concrete, and the autogenous shrinkage decreased with the increase in EA content. Notably, the 12% EA group showed volume expansion at 7 h, followed by gradual volume shrinkage, which decreased by 70.1% compared to control at 7 d. EA is affected by an alkaline environment during hydration, and CH crystals aggregated on surfaces of CaO, forming early swelling stress and improving the autogenous shrinkage of concrete [49]. However, excessive EA generated more CH crystals to cause early expansion. However, with the hydration of cement, the capillary negative pressure increased and the concrete shrank as a whole.

As shown in Figure 9b, with the addition of SRA, the autogenous shrinkage of concrete gradually decreases. Compared with the control, the autogenous shrinkage of concrete with 3% SRA is reduced by 79.6% at 7 d. This was mainly related to the structure of SRA molecule, which could reduce the surface tension of the capillary solution, thus reducing the development of negative capillary pressure in concrete [25,53].

It can be observed in Figure 9c that the autogenous shrinkage of concrete was significantly decreased after adding SAP, and it gradually decreased with the increase in SAP content [54,55]. The early free water exchange of SAP in cement-based materials was mainly controlled by osmotic pressure. Although this part did not play an obvious internal curing effect, it can increase the effective water-binder ratio of concrete. This is also the reason why the concrete with 0.4% and 0.6% SAP has less autogenous shrinkage in the first 3 d. After initial water release, SAP entered the second water release stage controlled by temperature and humidity, which delayed the reduction in the relative humidity in the concrete by continuously releasing water. Therefore, SAP played a better role in compensating for the autogenous shrinkage of concrete, and could even offset the autogenous shrinkage of concrete.

All three shrinkage-reducing components have significant effects on concrete autogenous shrinkage. Among them, SRA has the best effect on alleviating autogenous shrinkage and can be applied in secondary lining projects to achieve effective shrinkage reduction and reduce the generation of cracks.

### 3.5. Capillary Water Absorption

**Figure 10:**
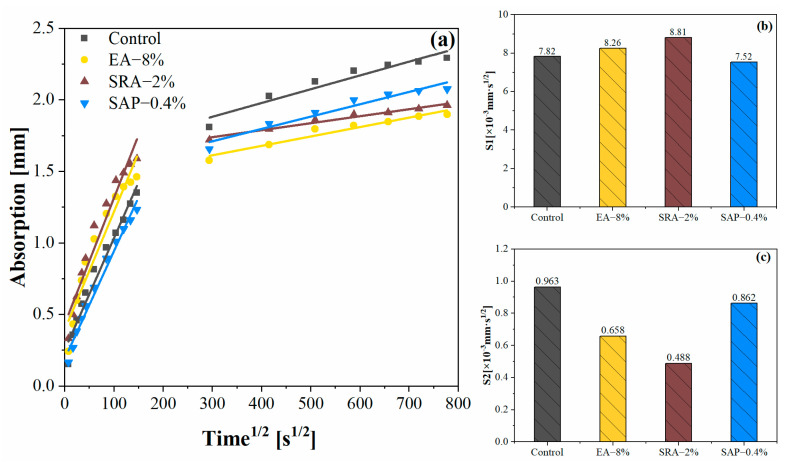
The different shrinkage-reducing components on the 28 d capillary water absorption of concrete: (**a**) total water uptake; (**b**) slope of S1 adsorption phase; (**c**) slope of S2 adsorption phase.

Capillary water absorption was used as a measurement of the ability of concrete to absorb liquid through capillarity. The test results were related to pore content and connectivity. Figure 10 shows the effect of different shrinkage-reducing components on the water transport of concrete at 28 d. The capillary water absorption was divided into two stages, based on the internal water transport mechanism of concrete [56]. The initial 6 h is the S1 stage controlled by the connectivity between capillary pores. 1–7 d is the S2 stage controlled by diffusive transport, where moisture fills mainly large and poorly connected pores.

As shown in Figure 10a, compared with control, the ability of EA and SAP groups to perform anti-capillary penetration was improved, and the improvement effect of EA is the most obvious. This is related to the improvement of EA on the internal porosity of concrete. In contrast, the delay of SRA on the hydration process of cement led to a decrease in the resistance to capillary penetration.

As shown in Figure 10b,c the initial adsorption coefficient S1 of EA-8% was lower than the control at 0–6 h. This is attributed to the formation of ettringite and calcium hydroxide formed by the hydration of EA, resulting in transformation of macropores into capillary pores. However, in the second stage, the adsorption coefficient S2 was 37.1% higher than the control, indicating that EA reduced the connectivity of the mortar capillary pores.

Compared with the control, the cumulative water absorption of SRA-2% and the adsorption coefficient in both stages were significantly improved, which was related to its higher internal pore content. During the cement hydration process, the of SRA makes negative effect, the loose cement matrix reduced the resistance of SRA-2% to moisture transport, which was confirmed by previous heat of hydration tests. Specifically, S1 and S2 increased by 12.6% and 22.5%, respectively, compared with the control [57].

The adsorption coefficient S1 in the first stage slightly decreased due to the effect of internal curing of SAP in cementitious materials [56]. On the one hand, SAP shrank and left macroscopic holes after completing the conservation action, which may lead to an elevation in S2. On the other hand, the repeated water absorption of SAP during the testing stage made it inevitably swell and block the permeation channel, hindering the capillary transport of water [58]. Overall, there is no significant change in the value of S2.

### 3.6. Chloride Permeability Test

**Figure 11:**
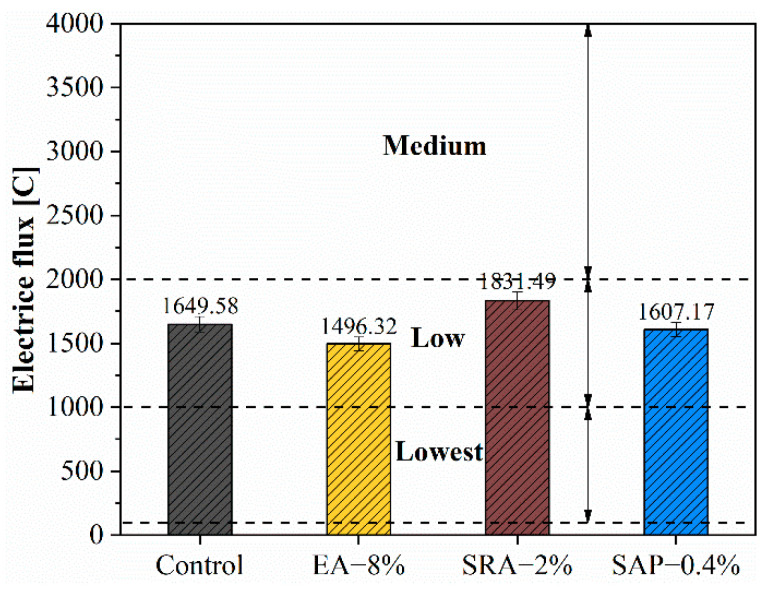
Influence of the electrical flux of concrete with the different shrinkage-reducing components.

Figure 11 shows the chloride penetration resistance of the concrete block with different shrinkage reducing components. It can be seen that EA is beneficial to chloride penetration resistance, and the electric flux of 8% EA is the lowest, which is 9.2% lower than the control. This was mainly attributed to the optimization effect of EA on the pore structure of concrete, which exerted expansion stress on the surrounding products through the space occupancy effect of CH crystals, reducing the passage of harmful ions. The addition of SRA increased the electric flux by 11% since that the SRA slows down the hydration process of the concrete [44], resulting in the increase in porosity and less dense structure in concrete, and promoting the transmission of harmful ions. The electric flux of the SAP group was almost identical to the control, only decreased by 2.5%. This was mainly affected by two aspects. On the one hand, SAP shrank into macroscopic pores after water release, which could provide channels for the transmission of chloride ions, and had a negative effect on the chloride penetration resistance of concrete. On the other hand, SAP could gradually release water to improve the hydration degree of cement interface transition zone and compact matrix structure in the later stage, which improved the chloride penetration resistance [59]. The combined action of the two reasons resulted in the electric flux of concrete with SAP being basically the same as control.

### 3.7. Pore Structure

**Figure 12:**
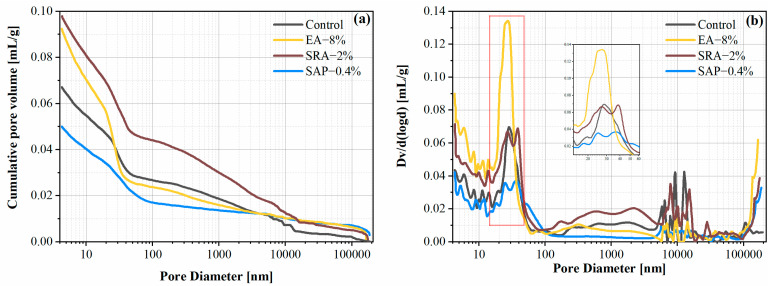
Influence of the different shrinkage reducing components on cement mortars: (**a**) the cumulative pore volume and (**b**) the pore size distribution.

Figure 12 shows the effect of the different shrinkage reducing components on the pore structure of the cement mortars at the age of 28 d. It can be visualized that the cement mortars mixed with SRA or EA has a higher cumulative pore volume, compared with the control. The matrix of the less hydrated cement is looser. The high pore volume of the SRA originates from the already mentioned inhibition of hydration by the SRA, which was confirmed in Section 3.2 and Section 3.3. It is noteworthy that the cumulative pore volume curve of EA-8% had a steep rise at the lower size pores, and the higher most-probable pore size peak of EA-8% was observed in the pore size distribution (Figure 12b) with a leftward shift compared with the other groups. This was associated with the fact that the EA has some fine holes encapsulated by the CH crystals during the expansion process. In addition, it was not unexpected that the internal curing effect of SAP admixture on the cement mortar reduces the volume of pores in the matrix.

Based on the pore size characteristics of cementitious materials, pores can be classified as gel pores (<10 nm), small capillaries (10 nm to 50 nm), medium capillaries (50 nm to 100 nm), large capillaries (100 nm to 10 μm), and macroscopic pores (>10 μm) [60,61].

The delineation results are shown in Table 4. The major pores in the EA group are gel pores and small capillaries, which account for 23.8% and 48.39% of the total volume, respectively. The percentage of large capillaries is reduced by 14.4% in EA-8% compared with the control, which indicated that EA significantly optimizes the pore structure of cement mortar. During the hydration of EA, the expansion of its volume blocked the large capillary pores, while the CH crystals carried some of the small pores. Moreover, CH and CaSO_4_ in EA promoted the secondary reaction of cement and mineral admixtures (GGBS, FA and SF) to generate AFt and fill the pores. The negative effect of SRA on the pore structure of the cement was again confirmed by the pore volume fraction. The percentage of gel pores and small capillaries of SRA-2% was reduced 5.1% and 10.6%, respectively, compared with the control. It is well known that the generation of gel pores and small capillary pores is bound up with the hydration of cement and mineral admixtures. The reduction in these two types of pores demonstrated that SRA exerts an inhibitory effect on cement hydration, which corresponds to the results in Section 3.3. Compared to the control, the SAP group showed a 15.62% decrease in large capillary pores and a 9.77% increase in macroscopic pores. The SAP produced more hydration products through continuous water release at the later stages of cement hydration, thus refining the pore size [59]. It is important to note that the addition of SAP optimized the capillary pores, yet SAP shrinkage left larger pores, leading to an increase in macroscopic pores.

## 4. Conclusions

In present work, the effect of different shrinkage-reducing agents on hydration rate and autogenous shrinkage of commercial concrete used in tunnel annular secondary lining engineering were investigated. The main conclusions are as follows:

1. EA, SRA, and SAP lead to different hydration heat release behaviors in commercial concrete. EA increases the degree of hydration and increases the total amount of exothermic heat of cementitious binder, while SAP and SRA have lower peaks and total hydration heat. EA, SAP, and SRA have a good compensation effect on autogenous shrinkage of commercial concrete. Among them, SRA-3% has the most obvious shrinkage reduction rate of 79.6%.
2. The negative effect of SRA and SAP on the early compressive strength of concrete is increased with the dosage. The compressive strength of SRA group is decreased by up to 44.6% at 3 d. However, the appropriate amount of SAP can equalize with the control group at 28 d. On the contrary, a moderate amount of EA has an increased effect on compressive strength. EA and SAP reduce the content of 100 nm–10 μm pores in commercial concrete by 49.5% and 54.2%.
3. The incorporation of EA can reduce the total capillary water absorption and improve the resistance to chloride ion penetration, while SRA has the opposite effect to EA. SAP group is close to the control group in terms of total capillary water uptake and resistance to chloride ion permeation.
4. The incorporation of EA can bring about better strength in the early stage and has the effect of improving the pore structure and enhancing durability performance. However, the addition of a large amount will produce an expansion effect and affect the volume stability of the concrete. SRA has an excellent ability to delay shrinkage, as well as the effect of reducing the rate of the heat of hydration, while it has a great influence on the mechanical properties. SAP compensates for the shrinkage with better late compressive strength and delays the hydration rate.
5. Compared with SRA and SAP, EA is more suitable for projects that require early strength and has better application value for secondary lining projects. SRA can effectively reduce the negative effects of concrete shrinkage, but the resulting decrease in strength will affect the subsequent duration of the secondary lining project. SAP as a new material applied to fill the shrinkage and anti-cracking still has more need for improvement. Under the premise of strict requirements for the compressive strength of concrete, SAP is a better choice for secondary lining projects with higher requirements for early hydration exotherm when choosing shrinkage reduction components.

## Figures and Tables

**Table 1:** Chemical composition of materials (%).

	MgO	Al_2_O_3_	SiO_2_	SO_3_	CaO	Fe_2_O_3_	P_2_O_5_	K_2_O	LOI
Cement	2.27	6.221	20.835	3.322	58.176	3.315	0.109	0.947	4.802
GGBS	7.45	15.707	31.58	3.813	39.313	0.289	0.029	0.398	4.079
SF	0.98	0.219	90.81	1.122	0.4	0.056	0.124	0.917	1.416
FA	0.63	41.343	42.479	1.215	5.34	3.719	0.362	0.827	5.37
EA	1.96	2.567	4.61	17.663	58.484	1.214	0.027	0.233	13.262

**Table 2:** Proportions of concrete mix (kg/m^3^).

Code	Cement	FA	GGBS	SF	Sand	Gravel	Water	EA	SRA	SAP
Control	270	150	75	20	860	880	175			
EA-4%	270	150	75	20	860	880	175	20.6		
EA-8%	270	150	75	20	860	880	175	41.2		
EA-12%	270	150	75	20	860	880	175	61.8		
SRA-1%	270	150	75	20	860	880	175		5.15	
SRA-2%	270	150	75	20	860	880	175		10.3	
SRA-3%	270	150	75	20	860	880	175		15.45	
SAP-0.2%	270	150	75	20	860	880	186.3			1.03
SAP-0.4%	270	150	75	20	860	880	197.6			2.06
SAP-0.6%	270	150	75	20	860	880	208.9			3.09

**Table 3:** Rheology parameters of compound pastes.

Sample ID	τ_0_/Pa	μ/Pa·s	Fitting Equation	R^2^
Cement	21.11257	0.82807	τ = 21.11257 + 0.82807γ − 1.29 × 10^−3^γ^2^	0.99318
EA-4%	10.42214	0.80191	τ = 10.42214 + 0.80191γ − 8.58388 × 10^−4^γ^2^	0.99759
EA-8%	10.69963	1.31995	τ = 10.69963 + 1.31995γ − 2.52 × 10^−3^γ^2^	0.99572
EA-12%	20.31382	1.48647	τ = 20.31382 + 1.48647γ − 3.08 × 10^−3^γ^2^	0.99617
SRA-1%	14.94641	1.03673	τ = 14.94641 + 1.03673γ − 1.39 × 10^−3^γ^2^	0.99748
SRA-2%	18.19536	0.787	τ = 18.19536 + 0.787γ − 1.11 × 10^−3^γ^2^	0.99657
SRA-3%	10.249	0.58992	τ = 10.249 + 0.58992γ − 7.8803 × 10^−4^γ^2^	0.99617
SAP-0.2%	27.09619	0.54142	τ = 27.09619 + 0.54142γ − 3.68807 × 10^−4^γ^2^	0.99902
SAP-0.4%	39.72358	0.51597	τ = 39.72358 + 0.51597γ + 5.06153 × 10^−6^γ^2^	0.99948
SAP-0.6%	39.88603	0.37507	τ = 39.88603 + 0.0.37507γ + 3.8843 × 10^−5^γ^2^	0.99893

**Table 4:** Pore volume and porosity of hardened mortars samples.

Samples	Porosity (mL/g)	Pore Volume Fraction (%)				
		<10 nm	10–50 nm	50–100 nm	100 nm–10 μm	>10 μm
Control	0.0671	18.25	38.6	3.36	29.05	10.74
EA-8%	0.0925	23.86	48.39	2.11	14.65	10.99
SRA-2%	0.0979	17.32	34.51	3.16	32.17	12.84
SAP-0.4%	0.05	19.45	37.41	9.2	13.43	20.51

## Data Availability

Not applicable.
